# Re-Audit on Improving Patients’ Experiences of Ward Round in Psychiatry Wards

**DOI:** 10.1192/bjo.2025.10629

**Published:** 2025-06-20

**Authors:** Rifat Binte Radwan, Chiro Islam Mallik, Harsha Gopishetty

**Affiliations:** Essex Partnership University NHS Foundation Trust, Chelmsford, Essex, United Kingdom

## Abstract

**Aims:** The re-audit aims to assess the improvements in patients’ experiences during ward rounds compared with the findings of the original 2022 audit.

It focuses on ensuring that all patients feel actively included in their ward rounds, fostering a more supportive and engaging experience.

The re-audit also examines whether patients’ physical health is given equal attention alongside their mental health and whether they are provided with appropriate and timely feedback regarding their progress during their inpatient stay.

**Methods:** The re-audit focused on 41 patients from Acute Adult Psychiatry, Psychiatry Intensive Care, and Perinatal Psychiatry Units, all of whom had participated in at least two ward rounds and had capacity to consent. Data was collected via face-to-face interviews with a questionnaire developed with input from the Patient Experience and Clinical Audit teams. The questionnaire used a modified Likert scale to improve clarity, ensuring confidentiality throughout the process. Standards referenced include NICE Guidelines (CG136) and Trust Policies.

**Results:** The re-audit highlighted several key improvements, including a higher percentage of patients receiving weekly consultant reviews, greater comfort speaking in front of professionals, an increased perception of attention to physical health, and a slight improvement in support provided before ward rounds. However, areas of concern emerged, such as decreased patient understanding of ward round discussions, worsened communication about schedule changes, lower satisfaction with the physical environment, and a higher percentage of patients feeling uninvolved in ward rounds.

**Conclusion:** Ward rounds are critical opportunities for service users to engage with professionals, seek clarity, and feel supported in their treatment journey. While the re-audit findings reflect some positive changes, particularly in consultant reviews, support, and physical health attention, there are clear gaps in communication, understanding, and inclusiveness. These areas are particularly critical in psychiatric wards, where effective communication and patient involvement are fundamental to care quality and outcomes. After analysing the re-audit results, finding out the root cause, focus on an improvement plan and implementing these plans accordingly and finally by monitoring the progress, there is a real potential to make this audit a quality improvement project.

